# Identification, Synthesis, and Characterization of N-Formyl Mirabegron: A New Degradation Product Formed by Formic Acid Impurity in Pharmaceutical Excipients

**DOI:** 10.1155/adpp/4971456

**Published:** 2024-12-10

**Authors:** Bashir Daoud Agha Dit Daoudy, Mohammad Ammar Al-Khayat, Ghassan Abo Chameh, Mohammad Amer Al Mardini

**Affiliations:** ^1^Department of Pharmaceutical Chemistry and Quality Control, Faculty of Pharmacy, Damascus University, Damascus, Syria; ^2^Department of Pharmaceutical Chemistry and Drug Quality Control, Faculty of Pharmacy, Arab International University (AIU), Ghabagheb, Syria; ^3^Department of Chemistry, Faculty of Science, Damascus University, Damascus, Syria; ^4^Department of Pharmaceutical Chemistry, Faculty of Pharmacy, Al Andalus Private University for Medical Sciences, Tartus, Syria

**Keywords:** compatibility tests, formic acid, mirabegron, N-formyl mirabegron, pharmaceutical excipients, reactive impurities

## Abstract

This study demonstrated the impact of formic acid (FAc), a common reactive impurity in pharmaceutical excipients, on the stability of mirabegron (MB). The investigation of MB-excipient compatibility tests revealed the formation of a new degradation product, FAc-DP, at 0.2% after 7 days of isothermal stress at 55°C. FAc-DP was synthesized and characterized as N-formyl MB using liquid chromatography–mass spectrometry (LC-MS) and both 1D and 2D nuclear magnetic resonance spectroscopy (NMR).

## 1. Introduction

Mirabegron (MB), a selective *β*3-adrenoceptor agonist, is the first agent of a new drug class for treating overactive bladder (OAB) syndrome. In 2012, the Unitied States Food and Drug Administration (U.S. FDA) approved MB extended-release tablets (Myrbetriq) for managing OAB symptoms in adults [1]. Later in 2021, MB for extended-release oral suspension (Myrbetriq Granules) was granted U.S. FDA approval for the treatment of neurogenic detrusor overactivity (NDO) in pediatrics aged 3 years and older [2].

**Figure 1:**
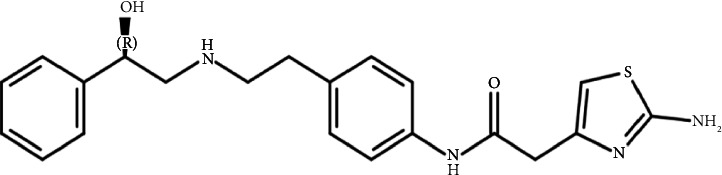
Mirabegron structure (R-enantiomer).

As shown in Figure 1, the MB structure includes hydroxyl and amino groups that can react with functional groups of many pharmaceutical excipients and/or their impurities which in turn may lead to form significant levels of degradation products (DPs) [3, 4].

**Figure 2:**
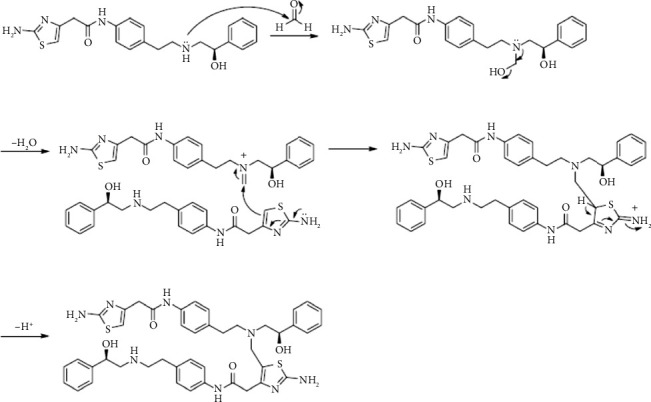
The postulated mechanism of MB degradation via Mannich reaction by Lin et al. [7].

Lin et al. [5] characterized the structure of a new DP (cyanomethyl MB) formed during the preparation of sample solutions of MB extended-release tablets for the analysis by high-performance liquid chromatography (HPLC). The formation of this DP resulted from the Strecker-like reaction in which the secondary amine group of MB reacted with trace amount of both formaldehyde (FA) in polyethylene glycol (PEG) and hydrogen cyanide in HPLC grade acetonitrile. Furthermore, Matsuzaki et al. [6] identified five DPs of MB during the accelerated stability tests of MB/solifenacin succinate bilayer tablets. The formation of these DPs was attributed to the reaction of MB amine groups with the aldehyde group of the acyclic forms of both disaccharide excipient (maltose) and its acidic hydrolysis product (glucose) via an acid-catalyzed Amadori rearrangement mechanism. Lin et al. [7] also demonstrated the formation of a novel DP during the stability studies conducted on MB extended-release tablets. The DP was characterized as methylene-bridged dimer of MB formed by the Mannich reaction between two molecules of MB and FA impurity in PEG and polyethylene oxide (PEO), as shown in Figure 2.

FAc and FA are common reactive impurities that can coexist in PEG and polyvinylpyrrolidone (PVP) excipients due to oxidative degradation. Both impurities can adversely affect the stability of drug products even in trace levels [8–12]. The influence of these impurities on the stability of drug products has been reported in many instances such as Benzocaine [13], Varenicline [14], Desloratadine, Saxagliptin [15], Pregabalin [16], and Naloxone [17].

Drug-excipient compatibility testing is a useful tool to predict potential stability problems and degradation pathways that can occur between the drug substance and the excipient itself and/or its impurities. However, the consistency and reproducibility of compatibility test results can be adversely affected by variations in the levels of FAc and FA impurities in pharmaceutical excipients from lot to lot and from vendor to vendor. These variations may be due to the complexity of establishing compendial limits that are suitable for the stability of all drug substances and products [10, 12, 18].

In this work, a compatibility test was carried out between MB and some excipients (PEG 6000, PEG 8000, and PVP K-30) that usually contain FAc and FA impurities. During the study, an unknown DP of MB (FAc-DP) was detected by HPLC along with methylene-bridged dimer of MB, identified previously by Lin et al. [7]. To our knowledge, FAc-DP is a novel impurity that has not been reported before.

The aim of this work is to demonstrate the role of FAc impurity in MB-excipient incompatibility as well as to identify, synthetize, and characterize FAc-DP.

## 2. Materials and Methods

### 2.1. Materials

MB (purity > 99%) was obtained from Saneca Pharmaceuticals (Slovak Republic). Excipients tested were PEG 6000, PEG 8000, and PVP K-30 (USP and BP grade). Chemicals and reagents used to conduct the study were as follows: FAc (≥ 98%, PanReac, European Union), FA solution (37%–40%, Surechem products, United Kingdom) acetic acid glacial (≥ 98%, PanReac, European Union), ethyl formate (EF) (≥ 99.5%, Fluka, Switzerland), tetrahydrofuran (THF) (≥ 99.9%, Sigma-Aldrich, United States of America), chloroform (≥ 99.9%, Sigma-Aldrich, Germany), absolute ethanol (≥ 99.8%, Honeywell, Germany), methanol (HPLC grade, Merck, Germany), methanol (LC-MS grade, Sigma-Aldrich, Germany), and ammonium acetate (≥ 98%, Scharlau, European Union).

### 2.2. Equipment and Instruments

Chemical reactions and drug-excipient compatibility tests were conducted using 10 mL glass vials equipped with polytetrafluoroethylene (PTFE)/silicone-lined screw caps (Agilent, the United States of America). Samples were incubated in UNE 400 oven (Memmert, Germany).

HPLC and LC-MS analyses were carried out by Shimadzu LC prominence system (Shimadzu, Japan) equipped with DGU-20A5 degasser, two LC/20AD pumps, CTO-20A column oven, SPD-20A UV/VIS detector, and LCMS-2020 single quadrupole MS detector.

Ultrapure water was produced by LaboStar TWF-UV 7 system (Siemens, Germany).

Analytical thin layer chromatography (TLC) experiments were conducted using aluminum plates precoated with silica gel 60 F_254_ (20 × 20 cm, layer thickness 0.2 mm, Merck, Germany), while preparative TLC isolation was performed by glass plates precoated with silica gel 60 F_254_ (20 × 20 cm, layer thickness 1 mm, Macherey-Nagel, Germany).

The synthesized FAc-DP was obtained using UNIVERSAL 320 R centrifuge (Hettich, Germany) and RE100-Pro digital rotary evaporator (DLAB, United Kingdom).

NMR spectra were recorded on Bruker Avance 400 UltraShield NMR spectrometer operating at 400.13 and 100.61 MHz for ^1^H and ^13^C, respectively.

### 2.3. Drug-Excipient Compatibility Study in Binary Mixtures

A solid-state compatibility study was performed on MB alone and in binary mixtures with excipients. MB (25 mg) and the excipients were weighed directly in 10 mL glass vials at a ratio (MB : excipient, w : w) of 1 : 1.5 for PVP K-30 and 1 : 5.8 for both PEG 6000 and PEG 8000. The mixtures were then vortexed for 1 minute and mixed thoroughly using a cut cap of 1.5 mL Eppendorf tube which was left inside the vial to prevent material loss. Each vial was then sealed and stored in the oven at a temperature of 55°C which was chosen because it is below the melting point of the excipients and not high enough to seriously impact the oxidative degradation during the period of study [15, 19, 20]. The sample vials were divided into two groups (*n* = 2 per sample in each group) which were analyzed after 7 and 21 days of incubation, respectively.

### 2.4. HPLC Method Development

The Shimadzu LC Prominence system was employed to develop the HPLC method for quantifying MB in the stressed samples. Key parameters, including mobile phase composition, flow rate, column temperature, and detection wavelength, were carefully examined to optimize chromatographic performance.

#### 2.4.1. Mobile Phase Preparation

The chromatographic separation was achieved using a mobile phase system consisting of two solutions: A (ammonium acetate buffer pH = 5 : methanol, 75 : 25, v/v) and B (ammonium acetate buffer pH = 5 : methanol, 30:70, v/v). Solution A was prepared by dissolving 9.784 g of ammonium acetate and 4 mL of acetic acid in 750 mL of water (pH = 5 ± 0.05). Then, 250 mL of methanol was added to the buffer solution and mixed thoroughly. Solution B was prepared in the same way but by adding 300 mL of water and 700 mL of methanol instead of 750 and 250 mL used in Solution A, respectively. The final buffer concentration in both solutions (A and B) was 200 mM.

The mobile phase was filtered through 0.45 *μ*m filters and degassed by sonication for about 20 minutes before use.

#### 2.4.2. Chromatographic Conditions

MB and its DPs were separated using Knauer C_8_ column (5 *μ*m particle size, 150 mm × 4.6 mm ID). The column oven temperature was maintained at 50°C. The detection wavelength was 250 nm, and the injection volume was 10 *μ*L. The gradient elution program was as follows: time (min)/mobile phase B (%): 0.01/0, 7/0, 10/1, 40/35, 45/40, 50/50, 55/75, 60/90, 62/100, and 72/100. Postequilibration was carried out for 10 min with 100% Solution A. The flow rate used throughout the run was 1.1 mL/min.

### 2.5. LC-MS

For identification of DPs, LC-MS analyses were performed on a single quadrupole LCMS-2020 mass spectrometer (Shimadzu, Japan). The MS was operated using atmospheric pressure chemical ionization source in positive mode (APCI^+^) with desolvation line (DL) temperature, 300°C; heat block temperature, 500°C; nebulizing gas flow, 1.5 L/min; scan speed, 1000 u/sec; event time, 1 s; detector voltage, 1.3 kV; interface voltage, 3 kV; DL voltage, 0 V; qarray direct constant (DC) voltage, 40 V; and qarray radio-frequency (RF) voltage, 40 V. The MS scans were recorded over the range of 100–1000 m/z.

The chromatographic separation was carried out utilizing the same mobile phase and conditions detailed in Sections 2.4.1 and 2.4.2.

### 2.6. HPLC Method Validation

The HPLC method was validated in terms of system suitability, specificity, linearity, accuracy, and precision. All solutions used to validate the method were prepared using Solution A as a diluent.

#### 2.6.1. System Suitability

The system suitability was determined by five replicate injections of MB solution at the test concentration of 500 ppm (25 mg/50 mL).

#### 2.6.2. Specificity

The specificity was evaluated by injecting separately the blank and the solutions of MB, stressed MB, excipients, and stressed excipients prepared individually in 50 mL volumetric flasks using the same quantities utilized in the preparation of binary mixtures.

#### 2.6.3. Linearity

The linearity study was established by preparing a series of MB solutions at five different concentrations: 400, 450, 500, 550, and 600 ppm which correspond to 80%, 90%, 100%, 110%, and 120% of the test concentration, respectively.

#### 2.6.4. Accuracy

The accuracy was estimated by recovery experiments at 100% of the test concentration using MB-excipient binary mixture samples (*n *= 3) prepared as described in Section 2.3. The samples were then dissolved and diluted to volume in 50 mL volumetric flasks.

#### 2.6.5. Precision (Intra- and Interday Precision)

Intraday precision (repeatability) was acquired by injecting MB solutions at 100% of the test concentration (*n = *6) on the same day and under the same experimental conditions, while interday precision (intermediate precision) was obtained by injecting the solutions in different days and by different analysts (*n = *12).

### 2.7. Preparation of the Stressed Samples for HPLC/LC-MS Analysis

The stressed samples were prepared for the HPLC/LC-MS analysis by dissolving the vial contents using Solution A as a diluent. The solutions were then transferred quantitatively to 50 mL volumetric flasks and diluted to volume with the same diluent. All sample solutions were injected directly after preparation.

### 2.8. Confirmation of FAc Role in MB Degradation

To confirm the responsibility of FAc for the formation of FAc-DP, a direct reaction between MB and FAc was performed as follows: 25 mg of MB was weighed directly in a 10 mL vial and dissolved with 300 *μ*L of methanol. Then, 50 *μ*L of FAc solution in methanol (16.05 mg/mL) was added. After that, the vial was sealed and incubated in the oven at a temperature of 55°C for 7 days.

### 2.9. Synthesis of FAc-DP

For FAc-DP synthesis, MB (74.7 mg) was dissolved in a mixture of 600 *μ*L of THF and 15 *μ*L of water. To this solution, 155 *μ*L of EF was added, and the mixture was stirred and heated at 55 ± 2°C for 17 h. The reaction progress was monitored by an analytical silica gel TLC plate using chloroform : absolute ethanol (9 : 1.95, v/v) as a mobile phase.

### 2.10. Isolation of the Synthesized FAc-DP by Preparative TLC

The synthesized FAc-DP was isolated and purified by a preparative silica gel TLC plate (1 mm in thickness) using the same mobile phase used for analytical TLC experiments. At the end of the development process, the TLC plate was removed from the chamber and dried at room temperature away from light. The target substance was then extracted from the relevant band using absolute ethanol. Subsequently, the ethanolic extract was centrifuged at a speed of 6000 rpm and a temperature of 5°C for 30 minutes. A vacuum evaporator was then used for evaporation of the extraction solvent at 50°C. Finally, 53.34 mg of a yellow solid substance was obtained with a reaction yield of 66.69%.

### 2.11. Structure Elucidation of FAc-DP

The structure of FAc-DP was confirmed using LC-MS, 1D NMR (^1^H and ^13^C), distortionless enhancement by polarization transfer (DEPT-135), and 2D NMR (^1^H-^1^H correlation spectroscopy [COSY-45] and heteronuclear single quantum coherence [HSQC]) using DMSO-d_6_ as a solvent at a concentration of ∼20 mg/mL. All NMR parameters are provided in the supplementary data (Figures S1–S7).

## 3. Results and Discussion

### 3.1. Development and Optimization of HPLC/LC-MS Method

The proposed method was developed and optimized by examining different conditions as discussed in Table 1.

### 3.2. HPLC Method Validation

The developed method was validated for system suitability, specificity, linearity, accuracy, and precision.  Table 2 summarizes the results of method validation.

All system suitability parameters were within acceptance criteria [21]: tailing factor (*T*) < 2, number of theoretical plates (N) > 2000, and relative standard deviation (RSD) of the peak area (*n* = 5) < 1%. The method was specific (Figures S8 and S9) and linear (*R*^2^ > 0.999) over the range of 400–600 ppm (Figure S10). Furthermore, the method was precise with an RSD < 2% both for intra- and interday precision. The accuracy was obtained from the recovery study. The recovery results obtained from all samples were between 98.5% and 101.9% with RSD values < 2%.

### 3.3. HPLC/LC-MS Analysis of the Stressed Samples

**Figure 3:**
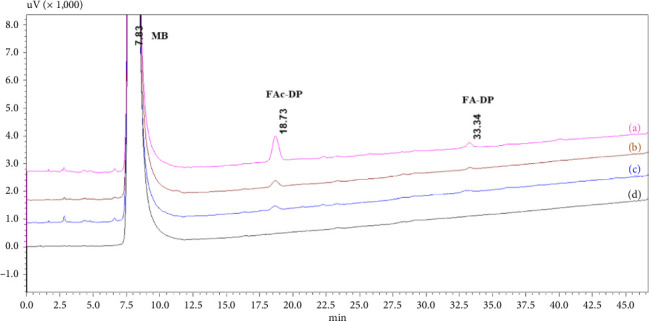
Overlayed HPLC chromatograms of MB-excipient stressed samples after 21 days of incubation at 55°C: (a) MB + PEG 8000, (b) MB + PVP K-30, (c) MB + PEG 6000, and (d) MB alone.

The stressed samples were directly analyzed by the HPLC/LC-MS method at the end of the incubation period. Overlayed HPLC-UV chromatograms (shown in Figure 3) revealed the formation of a major degradation product eluted at a retention time (*R*_*t*_) of 18.73 min with a relative retention time (RRT) of 2.39 (versus *R*_*t*_ of MB).  Table 3 shows that the percentage of FAc-DP was lower than 0.1% in PEG 6000 and PVP K-30 samples after 21 days of incubation. Otherwise, it was 0.2% in PEG 8000 samples after 7 days and increased to 0.34% after 21 days.

**Figure 4:**
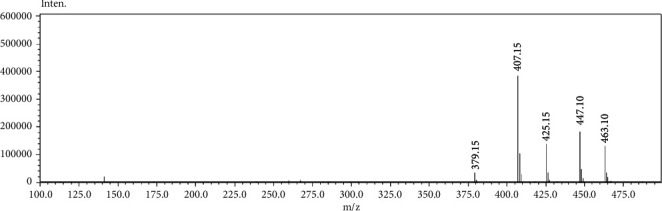
APCI^+^-MS spectrum of FAc-DP.

**Figure 5:**
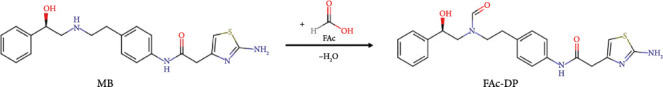
Reaction of MB with FAc.

The APCI^+^-MS spectrum of FAc-DP (Figure 4) shows the protonated molecular ion at 425 m/z with sodium and potassium ion adducts at 447 and 463 m/z, respectively. The mass difference between MB (396 dalton) and FAc-DP (424 dalton) by 28 dalton suggested that the degradation might be attributed to the reaction of MB with FAc. To confirm the validity of this hypothesis, a direct reaction between MB and FAc (Figure 5) was performed in the methanolic solution and analyzed by HPLC/LC-MS. The analysis' results demonstrated the formation of a FAc-MB adduct which eluted at the same *R*_*t*_ of FAc-DP and provided an identical mass spectrum. As a consequence, the formation of FAc-DP was due to FAc impurity present in the excipients, even though they met the requirements of the U.S. Pharmacopoeia (USP) and British Pharmacopoeia (BP).

**Figure 6:**
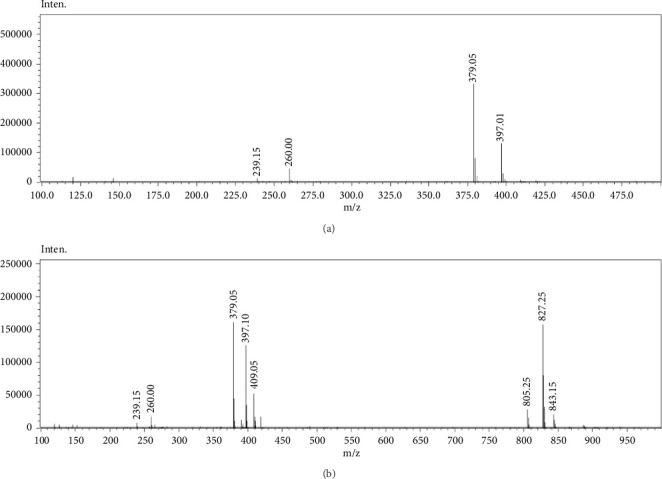
APCI^+^-MS spectrum of (a) MB and (b) FA-DP.

Figure 3 also shows another degradation product (FA-DP) detected at *R*_*t*_ of 33.34 min (RRT = 4.26). The percentage of FA-DP increased during the incubation but remained below 0.1%.  Figure 6 presents the APCI^+^-MS spectra for MB (a) and FA-DP (b). The spectrum of FA-DP indicates that the protonated molecular ion was at 805 m/z, with sodium and potassium ion adducts at 827 and 843 m/z, respectively. Furthermore, the characteristic ion at 409 m/z corresponded to iminium ion intermediate formed as the result of condensation of MB with FA. In addition, the fragmentation pattern of FA-DP matched that of the methylene-bridged dimer of MB observed by Lin et al. [7]. As a result, FA impurity in the excipients led to the formation of FA-DP. This was confirmed by performing a direct reaction between MB and FA in methanol.

### 3.4. Synthesis, Isolation, and Purification of FAc-DP

To obtain sufficient quantity for characterization, FAc-DP was synthesized by reaction of MB with EF in THF medium. Samples from the reaction mixture were diluted by THF and spotted onto the TLC plate. The FAc-DP was obtained at *R*_*f*_∼0.52.

The synthesized FAc-DP was isolated and purified by the preparative silica gel TLC technique. The HPLC/LC-MS analysis revealed that the *R*_*t*_ and MS spectra of the synthesized FAc-DP were identical to that of FAc-DP formed during the compatibility test, and the HPLC purity was 99.4% (by area normalization).

### 3.5. Structure Elucidation of FAc-DP

MB formylation may occur at three sites (the secondary alcohol, the secondary aliphatic amine, and the primary aromatic amine). To obtain the structure confirmation of FAc-DP, LC-MS, 1D and 2D NMR analyses were performed.

**Figure 7:**
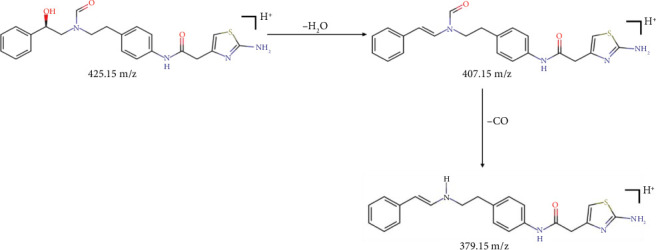
Proposed fragmentation pathway for FAc-DP.

Figure 7 shows the proposed MS fragmentation pathway for FAc-DP. The protonated molecular ion appeared at 425 m/z. The other ions observed at 407 and 379 m/z might correspond to the loss of H_2_O from the ion 425 m/z and CO from the ion 407 m/z, respectively. These results indicate that the molecular weight of FAc-DP is 424 dalton and that it is a formyl derivative. Furthermore, the presence of the fragment ion at 407 m/z indicates that the alcoholic group was not involved in the degradation reaction (Figure 5).

Table 4 summarizes the data analysis of 1D and 2D NMR spectra (Figures S1–S7) for FAc-DP which match the structure provided in Figure 8.

The 1D NMR spectra (Figures S1–S4) confirm the insertion of the formyl group at the secondary amine of MB. This was proved by the disappearance of the secondary amine proton signal (1.64 ppm, br, 1H) and by observing new proton (s, 1H) and carbon signals at chemical shifts of 7.85/7.75 and 163.25/162.99 ppm, respectively. The DEPT-135 spectrum (Figure S5) reveals that the new carbon signal corresponds to formyl CH. The signals of alcoholic (d, 1H) and aromatic amine (s, 2H) protons still present at 5.57/5.50 and 6.91, respectively, so that none of them was included in the degradation reaction.

NMR data also showed that the signals of carbons (6–22) and protons (7, 8, 10–15, 17–22 and OH) were duplicated. This can be explained by the existence of two isomers (cisoid and transoid) for the synthesized FAc-DP [22–24].

The 2D NMR (COSY-45 and HSQC) experiments were also employed to further confirm the structure of FAc-DP. The corresponding spectra (Figures S6 and S7) reveal that the considerable changes in the chemical shift occurred to protons and carbons located next to the reaction site (13 and 14), as shown in Table 4.

According to LC-MS and NMR data, the chemical degradation of MB by FAc impurity occurred at the secondary aliphatic amine, and N-formyl MB degradation product (FAc-DP) was formed.

## 4. Conclusion

In this research, MB degradation resulting from FAc reactive impurity was proved by studying its compatibility with PEG 6000, PEG 8000, and PVP K-30. The HPLC analysis revealed the formation of a new degradation product, FAc-DP, whose structure was confirmed as N-formly MB using LC-MS and NMR.

Due to the reactivity and prevalence of FAc in many pharmaceutical excipients, it is crucial to evaluate its potential interaction with other drug substances structurally similar to MB. This can help to exclude inappropriate excipients from early stage of formulation development, thus preventing the formation of undesirable DPs later.

## Figures and Tables

**Figure 8:**
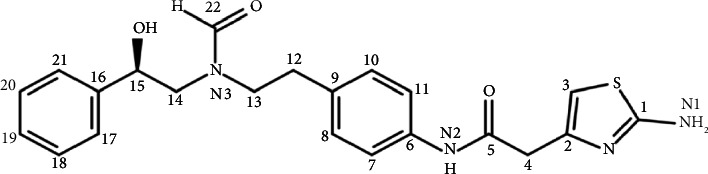
FAc-DP structure.

**Table 1:** Rationale for selecting HPLC/LC-MS conditions.

HPLC/LC-MS conditions	Selection rationale
Mobile phase composition	• Ammonium acetate/acetic acid buffer was used due to its volatility, making it suitable for subsequent MS detection
	• The buffer pH was tested from 4 to 6.8 • pH 5 provided optimal peak separation (resolution > 2) and suitable retention of MB
	• The buffer concentration in Solutions A and B was tested over the range of 50–200 mM • The final buffer concentration of 200 mM was convenient to achieve good separation and peak shape (tailing factor between 0.8 and 1.5)
	• Methanol was chosen as an organic modifier because it is compatible with the APCI^+^ mode

Column oven temperature	• The test range was 40°C–55°C • The column temperature of 50°C was adequate to obtain the desired resolution and peak symmetry

UV detection wavelength	• MB has two *λ*_max_ values at 207 and 250 nm. The detection wavelength was set at 250 nm, as it exceeds the UV cutoff of methanol and ammonium acetate

MS detection	• The APCI source is capable of handling volatile mobile phase buffers, evaporating mobile phases with high water content, and can tolerate flow rates up to 1.5 mL/min • The positive ionization mode is suitable for compounds containing amine groups • The voltages and temperature of the heat block were adjusted to give good intensities

**Table 2:** Summary of validation results.

Parameters	Results
Specificity	- No interferences or coelutions with the MB peak - Resolution between the MB peak and the nearest peak was 2.95

System suitability (at 100%) (*n* = 5)	Mean *T* = 1.27, mean *N* = 3563 RSD of the peak area = 0.44%

Linearity (80%–120%) (5 levels)	*R* ^2^ ≈ 1, slope = 17,622.2, *y*-intercept = 80,414.2

Precision (at 100%)	Repeatability: RSD = 0.59% (*n* = 6) Intermediate precision RSD = 0.65% (*n* = 12)

Accuracy (at 100%) (*n* = 3)	Mean recoveries: 100.3–100.8% RSD: 0.8%–1.7%

**Table 3:** FAc-DP percentage formed during the compatibility test of MB-excipient.

Incubation period (days)	FAc-DP (area %)			
	MB	MB + PEG 8000 (%)	MB + PEG 6000 (%)	MB + PVP K-30 (%)
0	ND	ND	ND	ND
7	ND	0.20	ND	0.04
21	ND	0.34	0.05	0.07

*Note:* The percentage of FAc-DP was calculated by area normalization.

Abbreviation: ND, not detected.

**Table 4:** NMR data of MB and FAc-DP.

#	MBa		FAc-DPc	
	^13^C *δ* (ppm)	^1^H *δ* (ppm)	^13^C *δ* (ppm)	^1^H *δ* (ppm)
1	167.83	N/A	167.84	N/A
2	145.91	N/A	145.84	N/A
3	102.61	6.30 (s, 1H)	102.58	6.30 (s, 1H)
4	N/Ab	3.46 (s, 2H)	39.76^d^	3.44 (s, 2H)
5	168.27	N/A	168.23	N/A
6	137.20	N/A	137.52/137.45	N/A
7	119.01	7.51 (d, 1H, *J* = 8.4)	119.00/118.94	7.52/7.51 (d, 2H, *J* = 8.4)
8	128.81	7.12 (d, 1H, *J* = 8.4)	129.11/128.85	7.14/7.09 (d, 2H, *J* = 8.4)
9	135.14	N/A	133.80/133.21	N/A
10	128.81	7.12 (d, 1H, *J* = 8.4)	129.11/128.85	7.14/7.09 (d, 2H, *J* = 8.4)
11	119.01	7.51 (d, 1H, *J* = 8.4)	119.00/118.94	7.52/7.50 (d, 2H, *J* = 8.4)
12	35.43	2.65 (m, 2H)	33.59/32.43^d^	2.73/2.71 (m, 2H)^e^
13	50.82	2.73 (m, 2H)	44.03/49.19^d^	3.51/3.33 (m, 2H)^e^
14	57.60	2.65 (m, 2H)	54.54/49.56^d^	3.32/3.26 (m, 2H)^f^
15	71.51	4.60 (m, 1H)	70.49/70.12	4.76/4.71 (m, 1H)^f,g^
16	144.66	N/A	143.55/143.01	N/A
17	125.89	7.22–7.32 (m, 5H, ArH)	126.07/125.99	7.26–7.36 (m, 5H, ArH)
18	127.93		128.07	
19	126.77		127.18/127.17	
20	127.93		128.07	
21	125.88		126.07/125.98	
22	N/A	N/A	163.25/162.99	7.85/7.75 (s, 1H)
N1	N/A	6.92 (s, 2H)	N/A	6.91 (s, 2H)
N2	N/A	10.02 (s, 1H)	N/A	10.03 (s, 1H)
N3	N/A	1.64 (br, 1H)	N/A	Disappeared
OH	N/A	5.25 (br, 1H)	N/A	5.57/5.50 (d, 1H, *J* = 4.6)^g^

^a^The signals were interpreted based on [7].

^b^C-4 signal overlapped with DMSO-d_6_ signal in ^13^C NMR.

^c1^H-^13^C correlations were determined by HSQC.

^d^The carbons were identified as CH_2_ by DEPT-135.

^e,f,g1^H-^1^H correlations recognized by COSY-45.

## Data Availability

The data used to support the findings of this study are available from the corresponding author upon request.

## Supporting Information

Figure S1: ^13^C NMR spectrum of MB (25 mg/mL). Figure S2: ^1^H NMR spectrum of MB (25 mg/mL). Figure S3: ^13^C NMR spectrum of the synthesized FAc-DP. Figure S4: ^1^H NMR spectrum of the synthesized FAc-DP. Figure S5: DEPT-135 spectrum of the synthesized FAc-DP. Figure S6: Expanded section of the COSY-45 spectrum of the synthesized FAc-DP focusing on the most important ^1^H-^1^H correlations. Figure S7: Expanded section of the HSQC spectrum of the synthesized FAc-DP focusing on the most important ^13^C-^1^H correlations. Figure S8: Overlayed HPLC chromatograms of unstressed MB and excipients: (A) MB, (B) PEG 6000, (C) PEG 8000, (D) PVP K-30, and (E) blank. Figure S9: Overlayed HPLC chromatograms of stressed MB and excipients at 55°C for 21 days: (A) MB, (B) PVP K-30, (C) PEG 8000, and (D) PEG 6000. Figure S10: Calibration curve of MB.
