# Supplementary material for: Identification, Synthesis, and Characterization of N-Formyl Mirabegron: A New Degradation Product Formed by Formic Acid Impurity in Pharmaceutical Excipients
Source: Adv Pharmacol Pharm Sci. 2024 Dec 10;2024:4971456. doi: 10.1155/adpp/4971456 (PMC11651750; doi:10.1155/adpp/4971456)
Supplement: Supporting Information — Additional supporting information can be found online in the Supporting Information section. [file 4971456.f1.docx]

**Supplementary Data**

**For**

**Identification, Synthesis and Characterization of N-Formyl Mirabegron: a New Degradation Product Formed by Formic Acid Impurity in Pharmaceutical Excipients**

Bashir Daoud Agha Dit Daoudy,^1^ Mohammad Ammar Al-Khayat,^2^  Ghassan Abo Chameh,^3^ and Mohammad Amer Al Mardini^1,4^

**Affiliations**

^1^Department of Pharmaceutical Chemistry and Quality Control, Faculty of Pharmacy, Damascus University, Damascus, Syria.

^2^Department of Pharmaceutical Chemistry and Drug Quality Control, Faculty of Pharmacy, Arab International University (AIU), Ghabagheb, Syria. Previously, Professor at the Faculty of Pharmacy, Damascus University.

^3^Department of Chemistry, Faculty of Science, Damascus University, Damascus, Syria.

^4^Department of Pharmaceutical Chemistry, Faculty of Pharmacy, Al Andalus Private University for Medical Sciences, Tartus, Syria.

**Figure S1:** ^13^C NMR spectrum of MB (25 mg/mL).

**Figure S2:** ^1^H NMR spectrum of MB (25 mg/mL).

**Figure S3:** ^13^C NMR spectrum of the synthesized FAc-DP.

Carbons at chemical shifts of 18.56 and 56.02 ppm correspond to residual ethanol, and carbon of chemical shift of 29.00 ppm corresponds to residual grease.

**Figure S4:** ^1^H NMR spectrum of the synthesized FAc-DP.

Protons at chemical shifts of 1.06 and 4.36 ppm correspond to residual ethanol, and protons of chemical shifts of 0.85 and 1.23 ppm correspond to residual grease.

**Figure S5:** DEPT-135 spectrum of the synthesized FAc-DP.


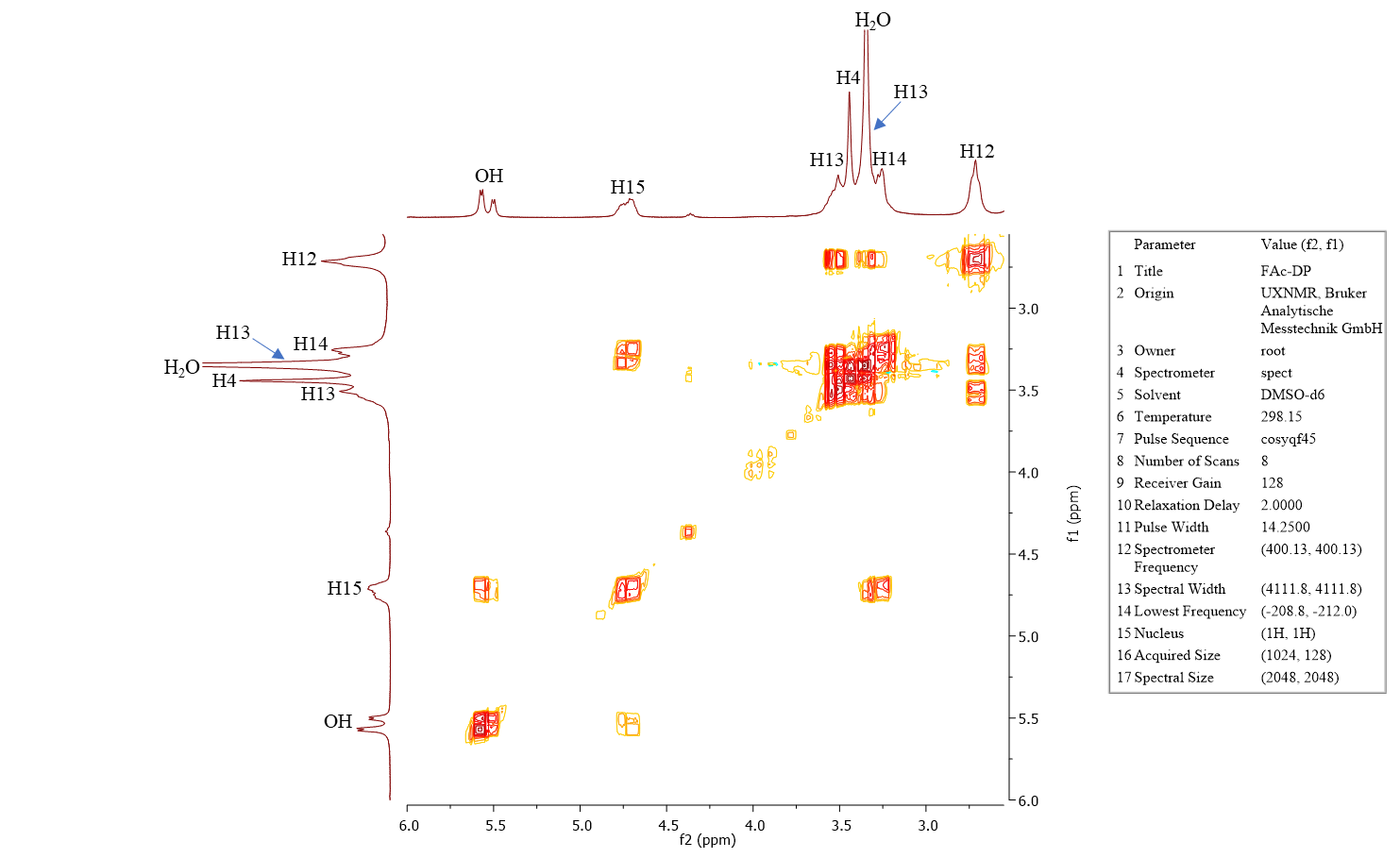


**Figure S6:** Expanded section of the COSY-45 spectrum of the synthesized FAc-DP focusing on the most important ^1^H-^1^H correlations.


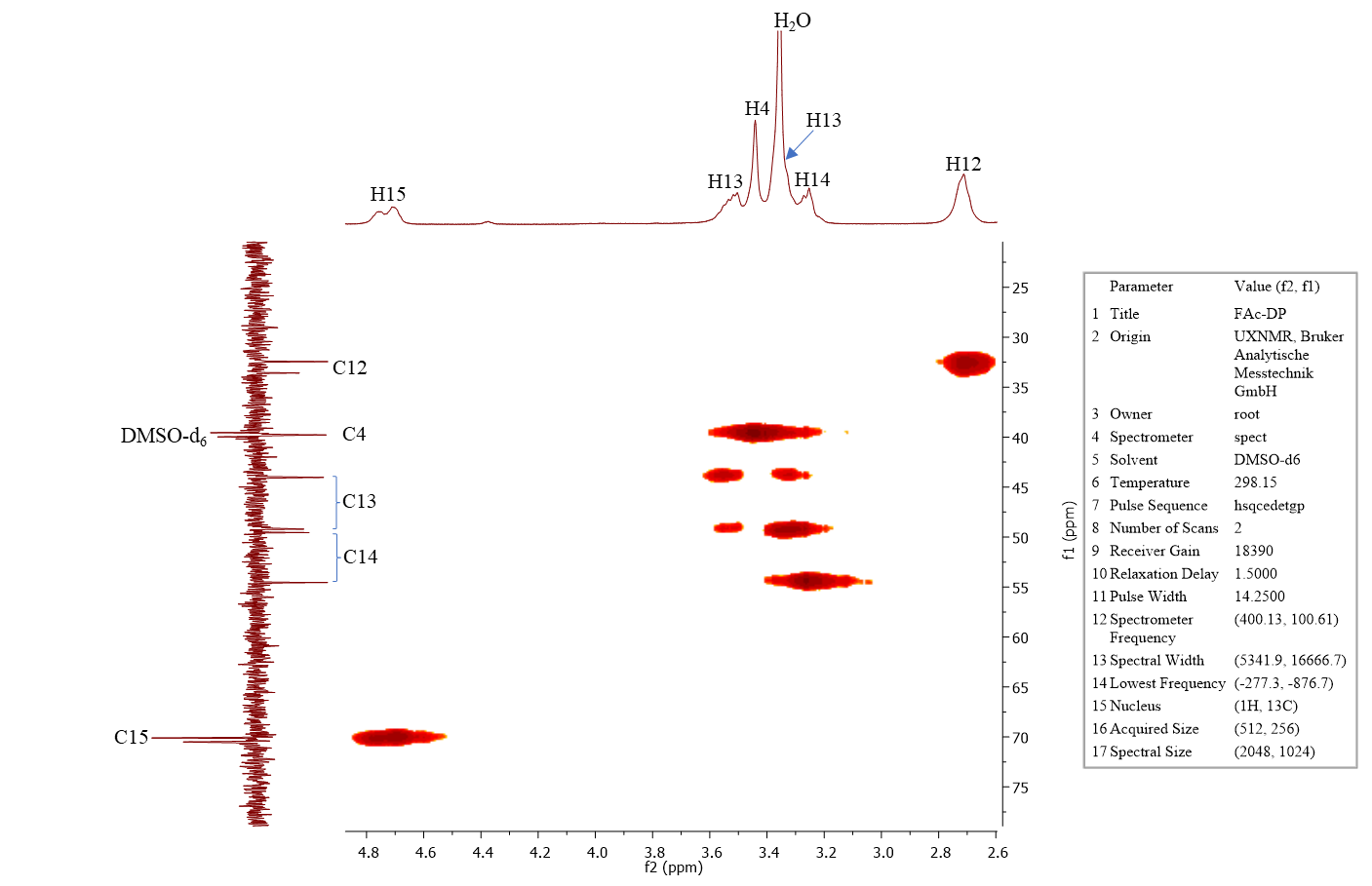


**Figure S7:** Expanded section of the HSQC spectrum of the synthesized FAc-DP focusing on the most important ^13^C-^1^H correlations.


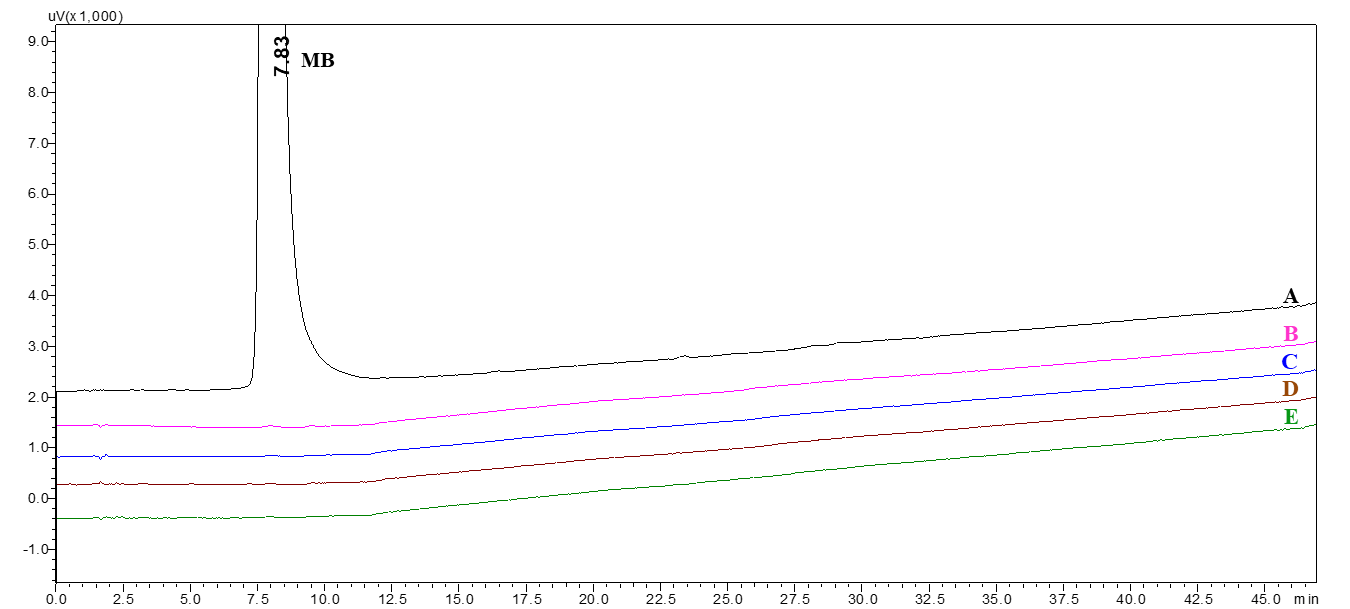


**Figure S8:** Overlayed HPLC chromatograms of unstressed MB and excipients: (A) MB, (B) PEG 6000, (C) PEG 8000, (D) PVP K-30 and (E) Blank.


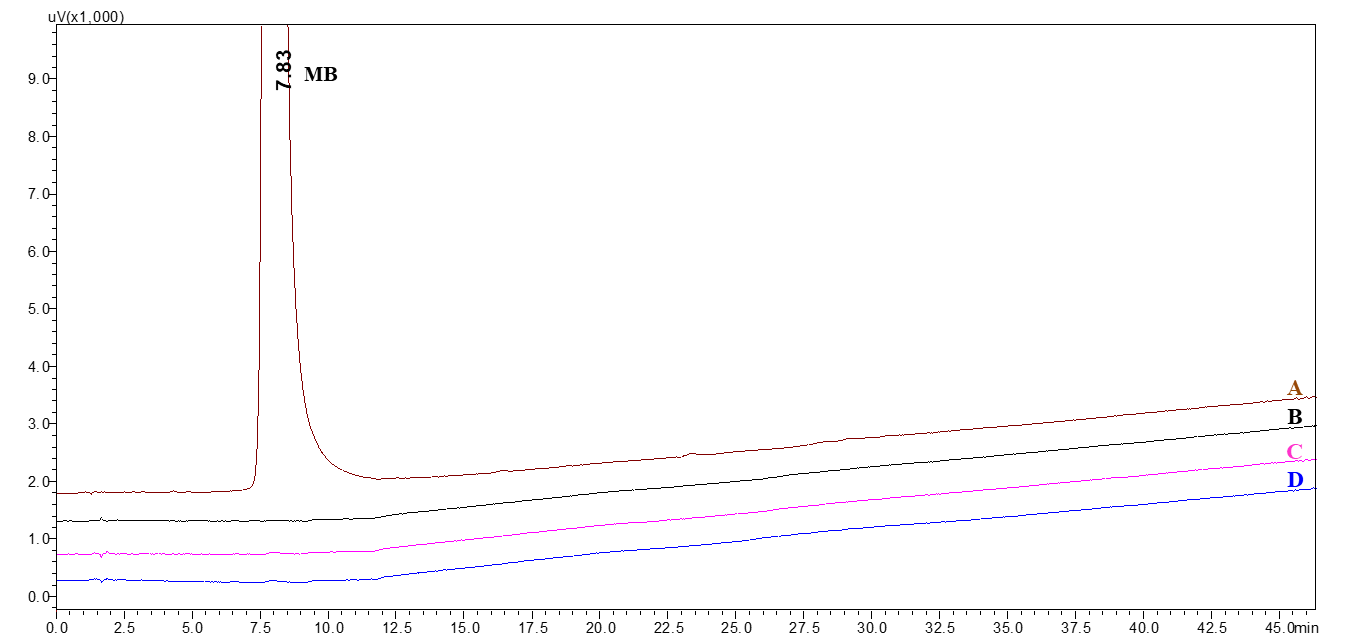


**Figure S9:** Overlayed HPLC chromatograms of stressed MB and excipients at 55℃ for 21 days: (A) MB, (B) PVP K-30, (C) PEG 8000 and (D) PEG 6000.

**Figure S10:** Calibration curve of MB.
